# Molecular Analysis of *CYP27B1* Mutations in Vitamin D-Dependent Rickets Type 1A: c.590G > A (p.G197D) Missense Mutation Causes a RNA Splicing Error

**DOI:** 10.3389/fgene.2020.607517

**Published:** 2020-11-27

**Authors:** Minjing Zou, Ayla Guven, Huda A. BinEssa, Roua A. Al-Rijjal, Brian F. Meyer, Ali S. Alzahrani, Yufei Shi

**Affiliations:** ^1^Department of Genetics, King Faisal Specialist Hospital and Research Centre, Riyadh, Saudi Arabia; ^2^Pediatric Endocrinology Clinic, Zeynep Kamil Women and Children Hospital, University of Health Science, Istanbul, Turkey; ^3^Department of Medicine, King Faisal Specialist Hospital and Research Centre, Riyadh, Saudi Arabia

**Keywords:** *CYP27B1* mutation, 1α-hydroxylase, vitamin D, rickets, RNA splicing

## Abstract

**Context:**

Vitamin D-dependent rickets type 1A (VDDR1A) is a rare autosomal recessively inherited disorder due to loss-of-function mutations in the *CYP27B1* gene. *CYP27B1* encodes an enzyme of 25-hydroxyvitamin D-1α-hydroxylase for converting inactive 25-OHD to biologically active 1,25-(OH)_2_D.

**Objective:**

To identify underlying genetic defects in patients with VDDR1A.

**Methods:**

Twelve patients from 7 Turkish and 2 Saudi families were investigated. The coding exons and intron-exon boundaries of the *CYP27B1* gene were amplified by Polymerase Chain Reaction (PCR) from peripheral lymphocyte DNA. PCR products were directly sequenced. The consequences of c.590G > A mutation were analyzed by *in silico* and functional analysis.

**Results:**

*CYP27B1* mutations were identified in all the patients. Two novel mutations were identified in two separate families: c.171delG (family 7) and c.398_400dupAAT (family 8). The intra-exon deletion of c.171delG resulted in a frameshift and premature stop codon 20 amino acids downstream from the mutation (p.L58Cfs^∗^20). The intra-exon duplication of c.398_400dupAAT generated a premature stop codon at the mutation site (p.W134^∗^). A missense c.590G > A (p.G197D) mutation was found in a patient from family 4 and caused a defect in pre-mRNA splicing. As a result, two populations of transcripts were detected: the majority of them with intron 3 retention (83%), and the minority (17%) being properly spliced transcripts with about 16% of wild-type enzymatic activity. The remaining nine patients from six families carried a previously reported c.1319_1325dupCCCACCC (F443Pfs^∗^24) mutation. Clinically, all the patients need continued calcitriol treatment, which was consistent with inactivation of 25-hydroxy vitamin D1α-hydroxylase activity.

**Conclusion:**

Two novel frameshift *CYP27B1* mutations were identified and predicted to inactivate 25-hydroxyvitamin D-1α-hydroxylase. The loss of enzymatic activity by c.590G > A missense mutation was mainly caused by aberrant pre-mRNA splicing.

## Introduction

Disorders in the biosynthesis of vitamin D result in vitamin D deficiency and can be classified into two groups: vitamin D-dependent rickets type 1A (VDDR1A, MIM 264700) and vitamin D-dependent rickets type 1B (VDDR1B, MIM 600081). VDDR1A and VDDR1B are caused by inactivation mutations in the *CYP27B1* gene (MIM 609506) and *CYP2R1* gene (MIM 608713), respectively (Acar et al., 2017).

Vitamin D is a group of biologically inactive pro-hormones. Its activation requires hydroxylation first in the liver where vitamin D is hydroxylated to 25-hydroxyvitamin D (25OHD) by 25-hydroxylase (CYP2R1) (Holick, 2007). The 25-hydroxyvitamin D is further hydroxylated in the kidney to 1,25(OH)_2_D by 25-hydroxyvitamin D-1α-hydroxylase. The biologically active 1,25(OH)_2_D binds to and activate vitamin D receptor to regulate calcium homeostasis and bone metabolism (Holick, 2007).

VDDR1A is a rare autosomal recessive disorder and is characterized clinically by growth retardation, hypotonia, hypocalcemic seizures, and rickets with typical laboratory findings such as hypocalcemia, elevated serum PTH, and low or undetectable level of serum 1,25(OH)_2_D despite normal or increased serum 25OHD (Fraser et al., 1973; Miller and Portale, 2003). Based on HGMD^1^, 79 different mutations have been reported from different ethnic groups (Kitanaka et al., 1999; Miller and Portale, 2000; Wang et al., 2002; Kim et al., 2007; Alzahrani et al., 2010; Demir et al., 2015).

In the present study, we investigated 12 VDDR1A patients from seven Turkish and two Saudi families.

## Materials and Methods

### Patients

Twelve patients from seven Turkish (Family 1–7) and two Saudi families (Family 8–9) were enrolled for the study. Their clinical and laboratory findings were summarized in Table 1. Clinically, the affected children had growth retardation, rickets, hypocalcemia, low levels of 1,25(OH)_2_D_3_ and elevated PTH. All of them required continued calcitriol treatment. The study was approved by the Institutional Review Board of King Faisal Specialist Hospital and Research Center and informed consent was signed by the guardian of the patients before enrollment.

**TABLE 1:** Clinical, laboratory, and genetic findings of patients with VDDR-1A rickets.

**Family**	**Patient**	**Age (M)**	**Clinical features**	***CYP27B1* mutation**	**Ca (mg/dl)**	**P (mg/dl)**	**ALP (IU/L)**	**25OHD_3_ (ng/mL)**	**1,25(OH)_2_D_3_ (pg/mL)**	**PTH (pg/mL)**
1	Father		Normal	Het carrier	ND	ND	ND	ND	ND	ND
	Mother		Normal	Het carrier	9.3	3.2	72	6.6	13.4	82.5
	Pt. 1	0–12	Poor feeding and failure to thrive	c.1319_1325dupCCCACCC F443Pfs*24, homo	8.9	2.4	199	37.9	22.8**	36.7
	Pt. 2	0–12	Unable to walk	c.1319_1325dupCCCACCC F443Pfs*24, homo	6.4	2.7	1,108	38.3	32.6	891.1
2	Father		Normal	Het carrier	9.8	3.2	85	26	17.2	48
	Mother		Normal	Het carrier	9.8	3.6	65	33.4	26.1	95
	Pt. 3	12–24	Growth retardation and unable to walk	c.1319_1325dupCCCACCC F443Pfs*24, homo	8.2	2.1	1,720	160*	32.9**	601
	Pt. 4	12–24	Growth retardation and leg bowing	c.1319_1325dupCCCACCC F443Pfs*24, homo	7.7	2.8	3,490	37	87**	ND
3	Father		Normal	Het carrier	9.9	3.4	61	20.2	14.1	51
	Mother		Normal	Het carrier	9.8	3.8	116	15.3	11.3	39
	Pt. 5#	0–12	Poor feeding and failure to thrive	c.1319_1325dupCCCACCC F443Pfs*24, homo	8.0	2.9	1,503	130*	21.28**	287
4	Father		Normal	Het carrier	9.5	3.3	62	19.9	ND	32.4
	Mother		Normal	Het carrier	8.7	3.9	75	10.1	ND	ND
	Pt. 6	0–12	Poor feeding and failure to thrive	c.590 G > A, p.G197D, homo	8.5	2	117	138*	42.1**	403
5	Mother		Normal	Het carrier	9.3	3.2	61	8.7	8.3	53.6
	Father		Normal	Het carrier	9.3	3	88	19.7	12.7	49.2
	Pt. 7	12–24	Unable to walk	c.1319_1325dupCCCACCC F443Pfs*24, homo	8.0	0.9	617	62	ND	642
	Pt. 8	0–12	Failure to thrive	c.1319_1325dupCCCACCC F443Pfs*24, homo	8.3	3.9	1,077	96.8	10.8	557.6
6	Mother		Normal	Het carrier	8.9	3.3	59	50.5	25.6	ND
	Father		Normal	Het carrier	9.8	3	108	17.9	12.1	25.8
	Pt. 9	12–24	Growth retardation and leg bowing	c.1319_1325dupCCCACCC F443Pfs*24, homo	7.6	2.9	1,424	305	5.9	367.1
7	Mother		Normal	Het carrier	ND	ND	ND	ND	ND	ND
	Father		Normal	Het carrier	ND	ND	ND	ND	ND	ND
	Pt. 10	12–24	Unable to walk	c.171delG, p.L58Cfs*20, homo, novel	6.6	3.0	1,161	28.2	15	495.7
8	Mother		Normal	Het carrier	ND	ND	ND	ND	ND	ND
	Father		Normal	Het carrier	ND	ND	ND	ND	ND	ND
	Pt. 11	12–24	Unable to walk	c.398_400dupAAT, p.W134*, homo, novel	7.0	3.6	1,984	17.8	14.5	670
9	Mother		Normal	Het carrier	ND	ND	ND	ND	ND	ND
	Father		Normal	Het carrier	ND	ND	ND	ND	ND	ND
	Pt. 12	12–24	Growth retardation and leg bowing	c.1319_1325dupCCCACCC F443Pfs*24, homo	8.2	3.9	1,870	20.4	12.4	458
Normal range					8.4–10.2	3.7–5.6	< 135	7–53	16–65	11–67

*Vitamin D3 300.000 unit/IM was given before test. **Under calcitriol treatment; ND: test is not done. ^#^Patient 5 is cousin of patients 3 and 4. Het, heterozygous; Homo, homozygous. SI unit conversions: to convert the values for 25OHD to nmol/L, multiply by 2.5; to convert the values for 1,25(OH)_2_D_3_ to pmol/L, multiply by 2.4; to convert the value for calcium to mmol/L, divide by 4; to convert the values for phosphate to mmol/L, divide by 3.1.

### Genomic DNA Isolation

Genomic DNA was isolated from peripheral blood lymphocytes using the Gentra Blood Kit (Qiagen Corp., CA).

### DNA Amplification and Sequencing

All coding exons and intron-exon boundaries of the *CYP27B1* gene were amplified by PCR from genomic DNA as described previously (Alzahrani et al., 2010). The resulting PCR products were directly sequenced using ABI PRISM 3700 sequencer (Foster City, CA).

### *CYP27B1* Mini-Gene Construction

To determine c.590G > A mutation on pre-mRNA splicing, we constructed a *CYP27B1* mini-gene by DNA synthesis, which contains exons 2–6 and their corresponding 150 bp introns from each splice donor and acceptor site if intron is more than 300 bp. The mini-gene sequence was shown in Supplementary Figure S1. The mini-gene was subcloned into pcDNA3.1 expression vector (Invitrogen, CA). The c.590G > A mutant was created by site-directed mutagenesis. Hek293 cells were cultured in DMEM/F12 medium containing 10% FBS and were transiently transfected with the mini-gene constructs. Total RNA was extracted 48 h after transfection as described previously (BinEssa et al., 2019).

### RT-PCR and Sequencing Analysis of *CYP27B1* Mini-Gene Transcripts

Two μg of total RNA were reverse-transcribed into cDNA using Promega reverse transcription system (Promega, Madison, WI). RT-PCR was performed to amplify the mini-gene transcripts using the following primer pairs: 5′-AACCCTGAACAACGTAGTCTG-3′ (in exon 3) and 5′-AACAGGAAGTGGGTCAGGTGC-3′ (in exon 5) for analysis of effect of c.590G > A mutation on pre-mRNA splicing. PCR conditions were 95°C for 5 min followed by 35 cycles of amplification (95°C for 1 min, 60°C for 1 min, and 72°C for 1 min) with final extension of 5 min. The PCR products were analyzed by agarose gel electrophoresis, and either directly sequenced or cloned into a TA vector (Invitrogen, CA) if multiple fragments were detected. Individual clones were subsequently sequenced.

### Measurement of 1α-Hydroxylase Activity

CHO cells were stably transfected with wild-type and c.590G > A mutant constructs as described previously (Alzahrani et al., 2010). Cells were seeded in 6-well plates overnight in growth medium followed by incubation in serum-free medium with 1μM saturating concentration of 25OHD_3_ (Sigma, MO) for 1 and 4 h, respectively. The 1,25(OH)_2_D_3_ in the medium was measured by radioimmunoassay (RIA) according to the manufacturer’s procedure (Immunodiagnostic Systems, AZ).

## Results

### Clinical Features

The clinical and biochemical features of patients were presented in Table 1. All the patients need continued calcitriol treatment. The clinical and laboratory features were consistent with inactivation of *CYP27B1* gene.

### Sequence Analysis of the *CYP27B1* Gene

**FIGURE 1:**
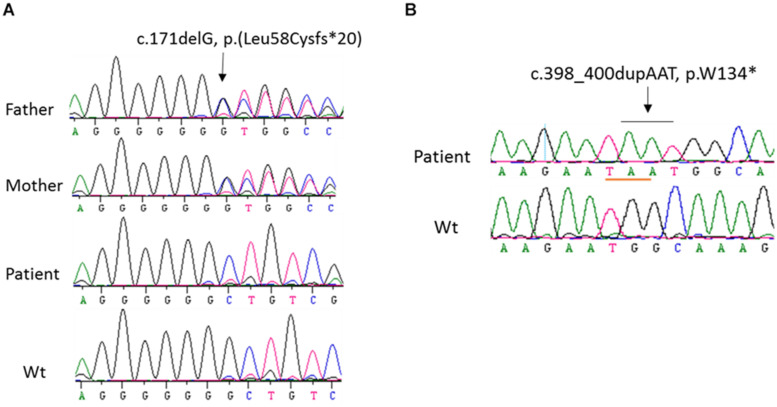
Sequence analysis of *CYP27B1* mutations. **(A)** A novel homozygous deletion of one nucleotide in the *CYP27B1* gene in patient 10 from family 7. The mutation results in frameshift and a pre-matured stop codon 20 nucleotides from the deletion. His parents carry a heterozygous mutation. **(B)** A novel homozygous duplication of AAT in the *CYP27B1* gene in patient 11 from family 10. The mutation creates a stop codon (highlighted in pink).

**FIGURE 2:**
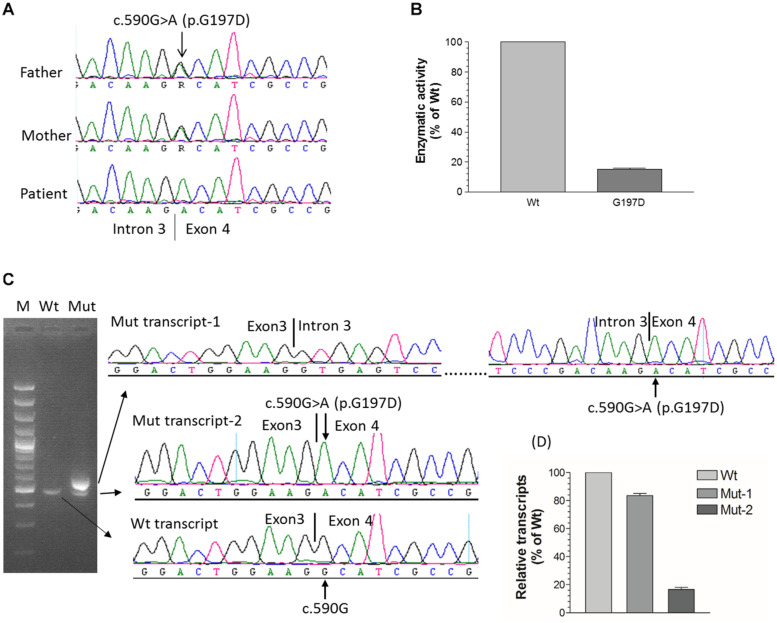
Functional characterization of c.590G > A (p.G197D) mutation. **(A)** The homozygous c.590G > A mutation in patient 6 from family 4, which is located at the first nucleotide of exon 4. Her parents carry a heterozygous mutation. **(B)** 1α-hydroxylase activity in CHO cells expressing wild-type and G197D *CYP27B1* cDNA. Enzymatic activity of G197D is expressed as a percentage of wild-type enzyme. The mutant retained 16% of wild-type1α-hydroxylase activity. **(C)** Impact of c.590G > A on pre-mRNA splicing by *CYP27B1* mini-gene analysis. Both wild-type and mutant constructs were transfected into Hek293 cells for *CYP27B1* mini-gene expression. RNA was isolated and reversed-transcribed to cDNA for RT-PCR and sequence analysis. The c.590G > A mutation leads to two populations of transcripts: retention of intron 3 (83% of transcripts from Mut-1) and correctly spliced transcripts (17% of transcripts from Mut-2). **(D)** Quantification of band intensity. The band intensity of **(C)** was quantified using ImageJ (https://imagej.nih.gov/ij/). Data are expressed as mean ± SEM.

In order to identify the inactivation mutations, the entire coding region and intron-exon boundaries of the *CYP27B1* gene were sequenced from the patients and their parents. All the patients were homozygous for the *CYP27B1* mutation and their normal parents were heterozygous carriers. Two novel mutations were identified: c.171delG in patient 10 from family 7 (Figure 1A) and c.398_400dupAAT in patient 11 from family 8 (Figure 1B). The c.171delG deletion resulted in a frameshift which created a premature stop codon 20 amino acids downstream from the mutation (p.L58Cfs^∗^20). The c.398_400dupAAT intra-exon duplication also created a premature stop codon at the mutation site (p.W134^∗^). The c.590G > A (p.G197D) mutation was found in patient 6 from family 4. Interestingly, the mutation was located at the first nucleotide of exon 4: at the junction of acceptor splice site of intron 3 (Figure 2A), which may affect RNA splicing. The mutation was predicted to be pathogenic by *in silico* analysis: disease-causing and likely to disturb normal splicing by Mutation Taster^2^ and possibly damaging with a score of 0.766 by PolyPhen-2.^3^ The remaining nine patients from six families were found to have previously reported mutations at c.1319_1325dupCCCACCC (F443Pfs^∗^24) (Supplementary Figure S2 and Table 1).

### Characterization of G197D Mutation on 1α-Hydroxylase Activity

Although c.590G > A was previously reported (Tahir et al., 2016), the functional consequence of the mutation on 1α-hydroxylase activity was not investigated. Given G197D was predicted to be pathogenic, we would like to study whether the mutant has any effect on 1α-hydroxylase activity. As shown in Figure 2B, during 1 h incubation with 25(OH)D_3_, CHO cells transfected with wild-type *CYP27B1* construct produced 285.6 ± 9.7 fmol/10^5^ cells of 1,25(OH)_2_D_3_ as compared to 46.9 ± 2.8 fmol/10^5^ cells transfected with G197D mutant. Thus, about 16% enzymatic activity were retained in the G197D mutant. Clinically, the patient responded to treatment very well. Initially, her weight and height standard deviation score (SDS) were −2.81 and −2.73, respectively. After treatment with calcitriol (60 ng/kg/day) and phosphate (55 mg/kg/day), her weight and height SDS became 1.42 and −0.45, respectively with normal serum Ca (9.5 mg/dL) and P (4.8 mg/dL). She is 9 years old now and currently under treatment with reduced dosage of calcitriol (16 ng/kg/day) and phosphate (9 mg/kg/day). Her last measurement of serum 25OHD_3_ was 42 ng/mL and 1,25(OH)_2_D_3_ was 42.1 pg/mL although serum Alp (380 IU) and PTH (125 pg/mL) were still elevated. The other patients also responded to treatment well but not as good as this patient.

### Mini-Gene Analysis of c.590G > A (p.G197D) Mutant

We next investigated effect of c.590G > A on RNA splicing. As shown in Figure 2C, the intron 3 was properly spliced out from wild-type *CYP27B1* mini-gene transcripts. Two populations of mutant mini-gene transcripts were detected: Mut-1 (83%) and Mut-2 (17%) (Figures 2B,C). The intron 3 was retained in the Mut-1 transcripts, leading to a frameshift and creation of a premature stop codon at I198Efs^∗^21. These data indicate that c.590G > A could severely interfere with the recognition by the splice machinery of canonical acceptor splice site in the intron 3, leading to intron retention. Although the intron 3 was properly spliced out in the Mut-2 transcripts which contain the c.590G > A (p.G197D) mutation, the translated protein have significantly reduced enzymatic activity.

## Discussion

In the current study, we have identified two novel mutations (c.171delG and c.398_400dupAAT) and two previously reported mutations (c.590G > A and c.1319_1325dupCCCACCC). The loss of enzymatic activity by c.590G > A missense mutation is mainly mediated by pre-mRNA splicing defect. Furthermore, c.1319_1325dupCCCACCC is found in six out of nine families, the most frequent mutation encountered in the study. The patients’ clinical and genetic features are consistent with *CYP27B1* inactivation and autosomal recessive inheritance.

The c.171delG and c.398_400dupAAT frameshift mutations have not been reported in other populations and they may be unique in the Turkish–Arab populations. The c.1319_1325dupCCCACCC has been reported in other ethnic groups and is most common insertion mutations found in the *CYP27B1* gene (Wang et al., 1998; Durmaz et al., 2012). Unlike other missense mutations, c.590G > A is located at the first nucleotide of exon 4 or at the junction of acceptor splice-site of intron 3, which may affect pre-mRNA splicing. In our previous studies, we reported a similar situation in the *CYP27B1* gene where a silent mutation (c.1215T > C, p.R379R) was found at the junction of donor splice-site (the last nucleotide of exon 7) (Demir et al., 2015). Although it was predicted to disturb normal pre-mRNA splicing by Mutation Taster, functional analysis did not demonstrate it could impair pre-mRNA splicing such as exon 7 skipping. The exon sequences surrounding the canonical donor splice-site GT or acceptor splice-site AG of each intron may contribute to different outcomes of these two mutations: G > A mutation at the first nucleotide of exon 4 (at the junction of acceptor splice-site) results in a splicing error whereas T > C at the last nucleotide of exon 7 (at the junction of donor splice-site) does not.

It has been reported that splice site recognition depends not only on its intrinsic strength (consensus vs. non-consensus sequences) but also on its context (Mount, 2000). Exon sequences at the splice junctions could affect fidelity of pre-mRNA splicing (Crotti and Horowitz, 2009; Reynolds and Hertel, 2019). They can function as a splicing enhancer by interaction with U1 snRNP (Watakabe et al., 1993). The recognition of an exonic splicing enhancer by the splice machinery plays a vital role in the selection of splice sites (Watakabe et al., 1993; Blencowe, 2000; Cáceres and Hurst, 2013). Burset et al. have developed a database of 22,489 known mammalian splice site sequences (SpliceDB) (Burset et al., 2001). Based on the SpliceDB, canonical GT-AG junctions are present at 98.71% and non-canonical GC-AG splice-site pairs at 0.56%. The consensus sequences and base-level frequencies extending the canonical donor splice-site GT (5′ splice-site) are A_6__0_G_80_| G_9__9_T_9__9_R_9__5_A_7__1_G_8__1_T_46_ (exon| intron, R is purine A or G). Guanine (G) at the last nucleotide of each exon occurs at 80% whereas A occurs at 9%, C 3%, and T 7%, respectively. The consensus sequences and base-level frequencies for acceptor splice-site (3′ splice-site) are C_7__1_A_9__9_G_99_| G_52_ (intron| exon). Guanine (G) at the first nucleotide of each exon occurs at 52% whereas A occurs at 24%, C 14%, and T 10%, respectively. The frequent appearance of G either at the last or first nucleotide of each exon indicates that G is relatively conserved at both 5′ and 3′ splice-sites. Given that c.590G is located at the first nucleotide of exon 4 (3′ splice-site) and is conserved, the G > A mutation would likely disrupt the recognition by the splice machinery to identify the correct acceptor splice site, resulting in intron 3 retention. On the other hand, the c.1215T is located at the last nucleotide of exon 7 (5′ splice-site) and is not conserved (G is the conserved nucleotide). The T > C mutation may not affect the recognition or base pairing between donor splice site of intron 7 and 5′ terminus of the U1snRNP in the early steps of splicing, thus having no effect on pre-mRNA splicing (Horowitz and Krainer, 1994). Although there are many reports showing missense mutations impair pre-mRNA splicing in different genes (Cartegni and Krainer, 2002; Yang et al., 2003; Aretz et al., 2004; Gonçalves et al., 2009), to our knowledge, this is the first report of aberrant splicing caused by a missense *CYP27B1* mutation.

Splice-site mutations or intra-exon insertion/deletions such as c.171delG, c.398_400dupAAT, and c.1319_1325dupCCCACCC often result in a shift of the open reading frame, leading to a premature stop codon and truncated proteins devoid of function or degradation of transcripts due to non-sense-mediated decay (Chang et al., 2007). In both situations, the enzymatic activity of 25-hydroxyvitamin D-1α-hydroxylase would be lost. Interestingly, 17% of c.590G > A-containing pre-mRNA transcripts can be properly processed and translated into protein. Although the mutant protein has only 16% of wild-type enzymatic activity, it may be sufficient to prevent development of more severe clinical phenotype such as hypocalcemic seizures and severe rickets. The good response to treatment with reduced calcitriol dosage support the residual enzymatic activity.

In conclusion, we have identified two novel frameshift mutations in the *CYP27B1* gene, which are predicted to inactivate *CYP27B1* gene. The c.590G > A missense mutation impairs pre-mRNA splicing.

## Data Availability Statement

The original contributions presented in the study are included in the article/Supplementary Material, further inquiries can be directed to the corresponding author/s.

## Ethics Statement

The studies involving human participants were reviewed and approved by the Office of Research Affairs, King Faisal Specialist Hospital and Research Center. Written informed consent to participate in this study was provided by the participants’ legal guardian/next of kin.

## Author Contributions

MZ and YS designed and analyzed the results and wrote the manuscript. AG and AA were involved in recruiting patients. HB, RA-R, MZ, BM, and AA were involved in acquiring data. All authors read and approved the final manuscript.

## Conflict of Interest

The authors declare that the research was conducted in the absence of any commercial or financial relationships that could be construed as a potential conflict of interest.

## Footnotes

1 https://portal.biobase-international.com/hgmd/pro/gene.php?gene=cyp27b1

2 http://www.mutationtaster.org/cgi-bin/MutationTaster/MutationTaster69.cgi

3 http://genetics.bwh.harvard.edu/pph2/index.shtml

## Supplementary Material

Supplementary Figure 1 *CYP27B1* mini-gene sequence (NM_000785). The c.590G > A mutation is highlighted in Bold.

Supplementary Figure 2 A previously reported homozygous duplication mutation in patient 1 from family 1.
