# Supplementary material for: Molecular Analysis of CYP27B1 Mutations in Vitamin D-Dependent Rickets Type 1A: c.590G > A (p.G197D) Missense Mutation Causes a RNA Splicing Error
Source: Front Genet. 2020 Nov 27;11:607517. doi: 10.3389/fgene.2020.607517 (PMC7729158; doi:10.3389/fgene.2020.607517)
Supplement: Supplementary Figure 1 — CYP27B1 mini-gene sequence (NM_000785). The c.590G > A mutation is highlighted in Bold. [file Data_Sheet_1.docx]

**Supplemental Material**

ATGGTGCAGGGCGCCGCGCACTTCGGGCCGGTGTGGCTAGCCAGCTTTGGGACAGTGCGCACCGTGTACGTGGCTGCCCCTGCACTCGTCGAGGAGCTGCTGCGACAGGAGGGACCCCGGCCCGAGCGCTGCAGCTTCTCGCCCTGGACGGAGCACCGCCGCTGCCGCCAGCGGGCTTGCGGACTGCTCACTGC (exon 2, 194 bp including ATG, ATG is added in front)

gtgagtcttctctgcccccagaagcccagacgccctgcagcggccctctcctgctgcgggtcccgaaactatcaatctgggggcatggtgggaggtcggctgtcccactctaccattgggcatcttggggttcccatcccagtctgcttccaggcgcaggccggtgcaggtttccgtaccccaagggggcagacgcaatccctctcctggccaccgcagccgaacgcgctccttcactgcagccagtcccgtcgagccgtccccaccttcccgatgcgcactctctcctcaaccctgcag (300 bp)

GGAAGGCGAAGAATGGCAAAGGCTCCGCAGTCTCCTGGCCCCGCTCCTCCTCCGGCCTCAAGCGGCCGCCCGCTACGCCGGAACCCTGAACAACGTAGTCTGCGACCTTGTGCGGCGTCTGAGGCGCCAGCGGGGACGTGGCACGGGGCCGCCCGCCCTGGTTCGGGACGTGGCGGGGGAATTTTACAAGTTCGGACTGGAAG (exon 3, 203 bp)

gtgagtcccaggacagagctgggcaggcgtcgggggcgccctaccagagcctcccggaaccctgacggcgccccctcccgacaag (85 bp)

**G**CATCGCCGCGGTTCTGCTCGGCTCGCGCTTGGGCTGCCTGGAGGCTCAAGTGCCACCCGACACGGAGACCTTCATCCGCGCTGTGGGCTCGGTGTTTGTGTCCACGCTGTTGACCATGGCGATGCCCCACTGGCTGCGCCACCTTGTGCCTGGGCCCTGGGGCCGCCTCTGCCGAGACTGGGACCAGATGTTTGCATTTG (exon 4, 201 bp, **c.590G>A, p.G197D**)

gtaaggcacaggtcgaggtggaaatgggggaatgtaaagctgtccaggggtagcgaggtattcacgtgccttctacccacgcag (84 bp)

CTCAGAGGCACGTGGAGCGGCGAGAGGCAGAGGCAGCCATGAGGAACGGAGGACAGCCCGAGAAGGACCTGGAGTCTGGGGCGCACCTGACCCACTTCCTGTTCCGGGAAGAG

TTGCCTGCCCAGTCCATCCTGGGAAATGTGACAGAGTTGCTATTGGCGGGAGTGGACACG (exon 5, 173 bp)

gtgaggttctccctccgtgctgtgagccggttccagggcttagcctccgcagactccggctccatttttctgttgcaggggatccattatggccacgtagaccagcttggcttagcaccctgtagccccagactcttccataatctgcaccctctgctgggttctcacacccaacacctctcttgctttcacatgtttttcag (203 bp)

GTGTCCAACACGCTCTCTTGGGCTCTGTATGAGCTCTCCCGGCACCCCGAAGTCCAGACAGCACTCCACTCAGAGATCACAGCTGCCCTGAGCCCTGGCTCCAGTGCCTACCCCTCAGCCACTGTTCTGTCCCAGCTGCCCCTGCTGAAGGCGGTGGTCAAGGAAGTGCTAAGATGA (exon 6, 177 bp)

Fig. S1. *CYP27B1* mini-gene sequence (NM_000785). The c.590G>A mutation is highlighted in Bold.


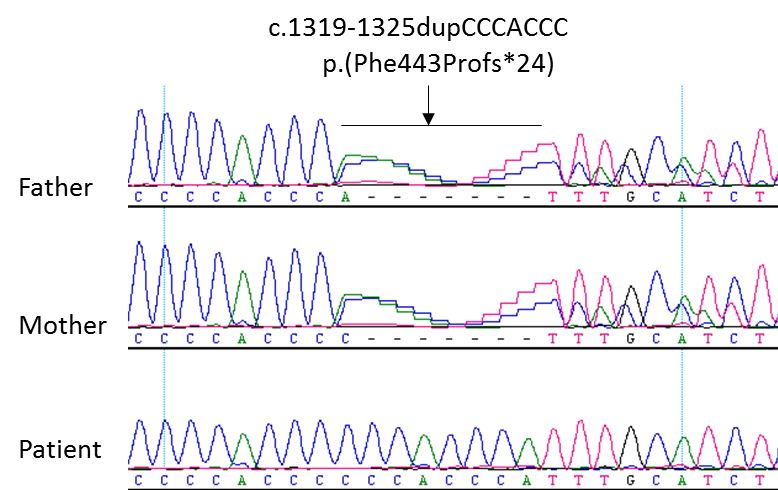


Fig. S2. A previously reported homozygous duplication mutation in patient 1 from family 1.
